# Editorial for the 1st Edition Special Issue “Benefits of Physical Activity and Exercise to Human Health”

**DOI:** 10.3390/sports14050183

**Published:** 2026-05-06

**Authors:** Ricardo Ferraz, Henrique P. Neiva, Fernanda M. Silva, Pedro Duarte-Mendes

**Affiliations:** 1 Department of Sport Sciences, University of Beira Interior, 6201-001 Covilhã, Portugal; hpn@ubi.pt; 2 Research Center in Sports Sciences, Health Sciences, and Human Development (CIDESD), 6201-001 Covilhã, Portugal; 3 School of Education and Communication, University of Algarve, 8005-134 Faro, Portugal; fmadsilva@ualg.pt; 4 Sport Physical Activity and Health Research & Innovation Center (SPRINT), 8005-134 Faro, Portugal; 5 Sport Physical Activity and Health Research & Innovation Center (SPRINT), 6000-084 Castelo Branco, Portugal; pedromendes@ipcb.pt; 6 Department of Sports and Well Being, Polytechnic Institute of Castelo Branco, 6000-084 Castelo Branco, Portugal

## 1. Introduction

Recent estimates indicate that the prevalence of insufficient physical activity has been increasing globally, with nearly one third of adults (31.3%) [1] and eight in ten adolescents (81%) [2] not meeting the World Health Organization physical activity guidelines (i.e., 150–300 min per week of moderate-to-vigorous intensity aerobic physical activity for adults and 60 min per day for children and adolescents [3]). According to authors [1,2], if the current trajectory continues, the global target of a 15% relative reduction in insufficient physical activity by 2030 [4] is unlikely to be achieved in most countries. This data are concerning given that, globally, 7.2% of all-cause deaths and 7.6% of cardiovascular disease deaths are attributable to physical inactivity [5].

Physical activity and exercise are widely recognized for promoting numerous health benefits, particularly in the prevention and management of obesity and cardiometabolic diseases [6,7], as well as in supporting immune function, reducing the risk of infectious diseases, improving mental health, and contributing to cancer prevention and control [8]. Moreover, regular engagement in these behaviors is associated with a reduced risk of all-cause mortality [9]. From a public health perspective, developing and implementing evidence-based strategies to increase physical activity and improve population health remains a global priority [8].

In this context, this Special Issue aims to advance knowledge on the role of physical activity and exercise interventions in promoting health and well-being across the lifespan. The contributions examine the effects of different exercise modalities on physical, cognitive, and mental health, as well as the role of physical activity in disease prevention and management and in improving functional capacity and quality of life across diverse populations. Collectively, the twelve papers included in this Special Issue provide complementary perspectives on these topics.

## 2. An Overview of Published Articles

Some studies in this Special Issue highlight the physiological and cognitive benefits of exercise interventions. The study by Nakao et al. (contribution 1) presents a randomized crossover trial examining the combined effects of aerobic training performed under mild hyperbaric oxygen (HBO) or normobaric normoxia (NN) environments, with concurrent eicosapentaenoic acid supplementation, on processing efficiency and interference indices in Stroop-type cognitive tasks among young men. The results showed improvements over time in processing efficiency following the training program, irrespective of the environmental condition. However, no additional cognitive benefit of HBO compared with NN was detected, and interference indices remained unchanged. In another study, Ramadan et al. (contribution 2) compared the hormonal responses in young women following a 10-week intervention of high-intensity interval training (HIIT) and traditional resistance training. Both exercise modalities were associated with significant increases in estrogen and reductions in testosterone, the follicle-stimulating hormone, and prolactin levels. Authors concluded that both exercise modalities may influence hormonal profiles, with potential implications for reproductive and metabolic health.

Other contributions in this Special Issue focused on the therapeutic potential of exercise interventions for the prevention and management of chronic health conditions. Moreales-Acuna et al. (contribution 3) demonstrated that a six-week boxing training program, performed three times per week, can be an effective and time-efficient strategy for improving vascular function and managing early stages of hypertension in young adults. Another study by Rad Bodan et al. (contribution 4) compared the effects of different eight-week intervention strategies on blood pressure, body mass index, and waist circumference in individuals with stage 1 hypertension, including healthy lifestyle recommendations (applied to all groups), antihypertensive medication (groups B, C, and D), a physical therapy program (group C), and hydrotherapy (group D). The results showed that interventions involving physical therapy and hydrotherapy resulted in greater improvements across these outcomes compared with lifestyle recommendations alone, even when patients needed to do programs without supervised support. Focusing on functional outcomes in older adults, Muanjai et al. (contribution 5) evaluated the effectiveness of an eight-week home-based stretching and strengthening training program for improving flexibility, muscle architecture, strength, and functional performance among individuals with leg tightness and/or suspected sarcopenia. The intervention improved flexibility, muscle strength, and functional performance, with strength-based exercises providing additional benefits, including muscle hypertrophy in knee extensors, suggesting that home-based exercise programs may represent a practical strategy to enhance physical function in older adults.

Additional contributions in this Special Issue explored the role of exercise interventions in clinical populations, with particular attention to mental health and well-being, as well as evidence-based exercise prescription. The study by Santos et al. (contribution 6) compared the effects of different supervised eight-week resistance training programs on mental health and well-being in individuals with spinal cord injury. Traditional resistance training showed the greatest improvements in anxiety, depression, and quality of life, whereas flywheel resistance training and high-velocity resistance training also produced beneficial, though more modest, effects. A scoping review by Peña et al. (contribution 7) aimed to characterize the load components of resistance training programs for kidney transplant recipients. The review highlighted the lack of standardized prescription criteria while providing evidence-based recommendations for training intensity, volume, and load progression that may serve as a reference for exercise professionals in designing resistance training programs for this population. Mena-Jiménez et al. (contribution 8) also conducted a scoping review on the return to physical activity in individuals with surgical stomas within the context of post-surgery rehabilitation. The authors found that current evidence is limited and heterogeneous, with most studies focusing on adult and oncology patients. The review highlights the need for more rigorous research and clearer guidance regarding the type, frequency, and intensity of exercise necessary to support safe participation in physical activity, avoid stoma-related complications, and improve quality of life in this population.

Other studies also explored the role of physical activity and sport in promoting motor development and psychological well-being in children and adolescents. Bekmanova et al. (contribution 9) compared the effectiveness of 12-week mini-handball training and conventional motor skill training (three sessions a week) on coordination in children with developmental coordination disorder. Results showed that both interventions improved coordination compared with the control group, although the mini-handball program produced greater improvements, particularly in tasks requiring anticipatory control and visuomotor integration. These findings seem to support the inclusion of sport-based, open-skill interventions in therapeutic and community programs, as well as in school curricula. The study of Di Martino et al. (contribution 10) raises another important question, aiming to evaluate whether physical activity and sports may act as protective factors in mitigating the long-term consequences of the COVID-19 pandemic in children and adolescents. Findings showed that four years after the pandemic, high percentage of anxiety and depression disorders remain among youth population. Sport participation appears to mitigate these effects, as adolescents with sport training experience showed lower depression rates.

Other studies also contribute to a better understanding of knowledge, performance, and safety in physical activity and sport settings. The study of Kobayashi et al. (contribution 11) conducted a cross-sectional survey with 1000 adults aged 20–69 years old to examine knowledge related to strength training, including the understanding of targeted muscle groups and proper exercise form. The results suggested that individuals with strength-training experience demonstrated higher knowledge across both domains, whereas those with instructional experience scored higher in knowledge of exercise form and movement. Lastly, the study of Berasategui et al. (contribution 12) described the physical responses of female football players with cerebral palsy during the 2022 Women’s World Cup and compared the responses according to the players’ sport class. Players classified as FT1 demonstrated lower physical responses during matches than those in FT2 and FT3, whereas similar performance profiles were observed between the FT2 and FT3 groups.

## 3. Concluding Remarks

Collectively, the studies included in this Special Issue reinforce the importance of physical activity and exercise across diverse populations and contexts. The contributions provide valuable insights into physiological responses to exercise, its role in the prevention and management of chronic conditions, and its potential to enhance functional capacity, motor development, and psychological well-being. Some studies also address practical aspects related to exercise prescription and safe participation in sport and physical activity, particularly in clinical populations.

Importantly, the presented evidence spans a range of research approaches, including experimental studies, randomized controlled trials, observational studies, and scoping reviews, and involves diverse populations from children and adolescents to adults, older adults, and clinical groups. This methodological and population diversity provides a broader perspective on current research and practice in physical activity and exercise.

Considering the high global prevalence of physical inactivity, these findings highlight the continued importance of research that supports safe, effective, and context-specific approaches to promoting physical activity and exercise across populations.

## Abbreviations

The following abbreviations are used in this manuscript:

HBO	Mild hyperbaric oxygen
NN	Normobaric normoxia
HIIT	High-intensity interval training

## References

[1] Strain T., Flaxman S., Guthold R., Semenova E., Cowan M., Riley L.M., Bull F.C., Stevens G.A., Country Data Author Group (2024). National, regional, and global trends in insufficient physical activity among adults from 2000 to 2022: A pooled analysis of 507 population-based surveys with 5·7 million participants. Lancet Glob. Health.

[2] Guthold R., Stevens G.A., Riley L.M., Bull F.C. (2020). Global trends in insufficient physical activity among adolescents: A pooled analysis of 298 population-based surveys with 1·6 million participants. Lancet Child Adolesc. Health.

[3] Bull F.C., Al-Ansari S.S., Biddle S., Borodulin K., Buman M.P., Cardon G., Carty C., Chaput J.P., Chastin S., Chou R. (2020). World Health Organization 2020 guidelines on physical activity and sedentary behaviour. Br. J. Sports Med..

[4] (2018). Global Action Plan on Physical Activity 2018–2030: More Active People for a Healthier World.

[5] Katzmarzyk P.T., Friedenreich C., Shiroma E.J., Lee I.M. (2022). Physical inactivity and non-communicable disease burden in low-income, middle-income and high-income countries. Br. J. Sports Med..

[6] Jakicic J.M., Behrens C.E., Deemer S.E., Forseth B., Katsanos C.S., Nickerson B.S., Prado W.L., Wang X., Deru L.S., Rogers R.J. (2025). Physical activity and exercise within the context of obesity treatment: Enhancing health beyond weight loss. J. Sport Health Sci..

[7] Belanger M.J., Rao P., Robbins J.M. (2022). Exercise, Physical Activity, and Cardiometabolic Health: Pathophysiologic Insights. Cardiol. Rev..

[8] Salvo D., Crochemore-Silva I., Wendt A., Tarp J., Shiroma E.J., Simpson R.J., Lee I.M., Ekelund U., Cerin E., Keita Y. (2026). Physical activity for public health in the 21st century. Nat. Med..

[9] Yu R., Duncombe S.L., Nemoto Y., Araujo R.H., Chung H.F., Mielke G.I. (2025). Physical activity trajectories and accumulation over adulthood and their associations with all-cause and cause-specific mortality: A systematic review and meta-analysis. Br. J. Sports Med..

